# ‘Dual Sensory Loss Protocol’ for Communication and Wellbeing of Older Adults With Vision and Hearing Impairment – A Randomized Controlled Trial

**DOI:** 10.3389/fpsyg.2020.570339

**Published:** 2020-11-26

**Authors:** Hilde L. Vreeken, Ruth M. A. van Nispen, Sophia E. Kramer, Ger H. M. B. van Rens

**Affiliations:** ^1^Department of Ophthalmology, Amsterdam University Medical Center, Vrije Universiteit Amsterdam, Amsterdam Public Health Research Institute, Amsterdam, Netherlands; ^2^Department of Otolaryngology, Head and Neck Surgery, Section Ear and Hearing, Amsterdam University Medical Center, Vrije Universiteit Amsterdam, Amsterdam Public Health Research Institute, Amsterdam, Netherlands; ^3^Department of Ophthalmology, Elkerliek Hospital, Helmond, Netherlands

**Keywords:** vision disorders, hearing disorders, deaf-blind disorders, dual sensory impairment, communication, quality of life, aged, communication partner

## Abstract

**Objectives:**

Many older adults with visual impairment also have significant hearing loss. The aim was to investigate the effectiveness of a newly developed Dual Sensory Loss (DSL) protocol on communication and wellbeing of older persons with DSL and their communication partners (e.g., spouse or child) in the Netherlands and Belgium.

**Methods:**

Participants (*N* = 131) and their communication partners (*n* = 113) were randomized in the “DSL-protocol” intervention group or a waiting-list control group. The intervention took 3 to 5 weeks. Occupational therapists focused on optimal use of hearing aids, home-environment modifications and effective communication strategies. The primary outcome was the Communication Strategies domain of the Communication Profile for the Hearing Impaired (CPHI). Secondary outcomes measured in participants were the Low Vision Quality Of Life Adjustment subscale, the Center for Epidemiological Studies - Depression Scale, De Jong Gierveld Loneliness Scale and the Fatigue Assessment Scale. The Hearing Handicap and Disability Inventory (HHDI) - Reaction of Others subscale and the Care-related Quality of Life - 7 Dimensions was measured in communication partners. Measurements were taken at baseline and 3-month follow-up. Linear mixed models (LMM) were used to analyze effects between groups over time for every outcome measure.

**Results:**

Intention-to-treat analyses showed a significant effect of the DSL-protocol on the use of verbal strategies (effect size SMD = 0.60, 95% CI: 0.25 to 0.95) in favor of the control group, however, this effect was non-significant after adjustment for confounding. Effect sizes of other outcomes varied between −0.23 [−0.57, 0.12] and 0.30 [−0.05, 0.64]. The LMM showed a significant effect on the HHDI-Reaction of others scale in favor of communication partners in the treatment group, however, the effect did not remain significant at a 0.01 significance level and the effect size was very small and non-significant 0.12, 95% CI [−0.27 to 0.51]. Adjusted analyses did not reveal treatment effects.

**Conclusion:**

The DSL-protocol did not clearly contribute to the enhancement of communication and wellbeing in DSL-patients. Possible reasons for the lack of effects are OTs not being comfortable giving advice on communication and psychosocial issues or the short-term treatment and follow-up period. Further study is warranted to find out how the protocol may be adapted or whether it is necessary to involve mental healthcare professionals.

**Clinical Trial Registration:**

www.ClinicalTrials.gov, identifier NTR2843.

## Introduction

Communication and social interaction are important for everyone. Proper hearing and vision are essential for effective communication. However, among older adults, vision loss, hearing loss and also DSL are relatively common (Schneider et al., 2012; Vreeken et al., 2014; Guthrie et al., 2016). Age-related hearing loss significantly reduces speech discrimination ability. Use of visual cues such as seeing the speaker’s face and interpretation of gestures improves speech perception substantially. In case of DSL, however, visual impairment may significantly hamper speech-reading ability of patients involved (Dickinson and Taylor, 2011; Morris et al., 2012). Missing out on both audiological as well as visual information exacerbates communication difficulties and social interaction (Jang et al., 2002). This may result in loneliness, social isolation, dependence, and in turn, may lead to reduced quality of life and social participation (Crews and Campbell, 2004; Heine and Browning, 2004; Kiely et al., 2013; McMahon et al., 2017; Jaiswal et al., 2018). Studies have shown associations between DSL and loneliness (Savikko et al., 2005) or depression (Huang et al., 2010; Yamada et al., 2014). Also, DSL-patients are more prone to experience a breakdown in communication, which is known to evoke negative feelings such as anger and frustration (Heine and Browning, 2004). In addition, persistent fatigue takes a considerable toll on DSL-patients. Due to the intense concentration required during listening and seeing, the effort needed for communication in daily life consumes energy and exerts on physical strength (Heine and Browning, 2004; Yamada et al., 2014).

Despite the well-known positive effects of hearing aids (HA) (Ciorba et al., 2012), amplification is often not sufficient to optimize communication in daily life listening situations, such as in group conversations or when there is background noise. In addition to HA fitting, communication strategies such as reducing background noise, lip reading and verbal or non-verbal strategies, may further improve communication. In audiological rehabilitation, there are communication protocols which aim to teach patients with hearing impairment to use adequate communication strategies (Hickson and Worrall, 2003; Chisolm et al., 2004; Kramer et al., 2005). However, often audiological en vision rehabilitation services are delivered separately. Moreover, audiological services usually do not take concurrent vision impairment into account and researchers explicitly exclude patients with DSL (Chisolm et al., 2004).

There are a few initiatives reported in the literature to integrate healthcare for older adults with DSL (McMahon et al., 2017; Roets-Merken et al., 2018). McMahon et al. (2017) performed an audiological screening of older adults with low vision and provided education and rehabilitation to those with hearing loss who did not own or did not regularly use hearing aids. However, the hearing handicap did not differ between persons seeking help and those not seeking help by a hearing aid provider. Roets-Merken et al. (2018) developed a nurse-supported self-management program for older adults with DSL in long-term care homes. The self-management program affected the domain ‘instrumental activities of daily living’ of social participation; however, the other outcomes showed no effect of the program. Problems in communication do not only affect the DSL-patient, they affect everyone with whom the patient communicates on a regular basis and can develop into so-called third-party disability (Hickson et al., 2007). In the audiological literature, a communication partner is defined as a person with whom an important and regular relationship is maintained, i.e., a person who frequently interacts with the person with hearing loss. The communication partner is often the spouse or partner, but can also be a child, friend, or neighbor (Kamil and Lin, 2015). Communication-partners play an important role in overcoming communication problems. However, communication partners are often unaware of the difficulties experienced by the DSL-patient (Heine et al., 2002). Research has shown that involvement of communication partners in rehabilitation increases awareness, receptiveness and motivation for making adaptations in attitudes and behavior (Hallam et al., 2008; Manchaiah et al., 2013).

In this study, a comprehensive DSL-protocol was developed and applied by occupational therapists (OT) working in low vision services in the Netherlands and Belgium (Vreeken et al., 2013). The main objective of the DSL-protocol is to increase communication ability and to improve wellbeing among persons with DSL. The protocol offers guidance to OTs to focus on optimal use of remaining vision and hearing with HAs and assistive devices, on home-environment modifications, and on the use of effective communication strategies. The DSL-protocol proved to be beneficial regarding HA utilization, but only in a subgroup of patients who experienced difficulties with their HAs; no significant overall effect on HA use was found (Vreeken et al., 2015). The main objective of the present study was to evaluate the effectiveness of the DSL-protocol on communication of DSL-patients. Secondary aims were to investigate the effectiveness of the protocol on wellbeing of patients and communication partners, and also the partners’ attitudes toward hearing loss.

## Materials and Methods

### Research Design

A randomized controlled trial was conducted to examine the effectiveness of the DSL-protocol by comparing the outcomes of the intervention group to a waiting-list control group. All participants had completed regular low vision and audiological services. The trial was performed in 2013 in regional centers of three multidisciplinary low vision services in The Netherlands and Belgium (Flanders). For practical reasons, randomization was stratified per OT’s area of practice (eight strata), to ensure equal distribution of participants in each of these areas. Data were collected in structured face-to-face interviews at the participants’ homes by trained research assistants at baseline and at 3 months follow-up. After completion of baseline measurements, an independent researcher not involved in the trial assigned participants in blocks of two to each stratum using randomization software. Participants were informed about their allocation by regular mail. Participants were not masked. The research assistants who performed the outcome assessments were masked regarding group allocation (participants were asked by the research assistant to conceal allocation at follow-up), as was the investigator performing the data analyses. OTs were only notified which participants they had to offer treatment according to the DSL-protocol. To avoid contamination, OTs were neither aware which participants were in the control group, nor did they have any contact with them. Participants in the control group were offered treatment after completion of the follow-up measurement.

### Intervention

The DSL-protocol consisted of a comprehensive handbook with background information and a checklist with exercises. OTs who had received a 1-day training, delivered the DSL-protocol to participants with DSL and their communication partners in three to five weekly sessions at the participant’s home. The protocol consisted of: (1) optimal use of HAs; (2) use of assistive devices and adaptations to the living environment; and (3) coping with DSL and use of effective communication strategies. The third part of the DSL-protocol was based on the home education program for older adults with hearing impairment developed by Kramer et al. (2005). It aims to raise awareness of communication problems between patient and partner and addresses adequate coping, including the use of effective communication strategies in order to enhance communication. Examples of communication strategies are: reducing background noise, shortening the distance to the communication partner and reducing glare, or conversational strategies such as explanation of sensory impairments to the communication partner, use of clarification or repetition of requests, and asking the communication partner to speak slowly or to articulate well.

Occupational therapists showed participants and/or their communication partners a short film which presented staged cases of effective and ineffective behaviors of both a hearing impaired person and the communication partner(s) and showed how use of effective communication strategies could improve communication (Kramer, 2008). Based on this short film, OTs discussed coping behaviors and communication strategies with participants and their communication partners. In addition, they gave a large-print hand-out to the participant and communication partners with suggestions on how to improve communication. Furthermore, the OTs discussed relevant issues concerning coping with DSL such as an individual’s energy balance, fatigue and peer support. More details of the DSL-protocol are described elsewhere (Vreeken et al., 2013).

### Sample Size and Ethical Review

Participants were recruited through low vision services in the Netherlands and Belgium. Inclusion criteria with respect to vision corresponded to the eligibility requirements for low vision services. Criteria for eligibility for low vision services are described in the guideline “Vision disorders, rehabilitation and referral” of the Dutch Society of Ophthalmology (NOG) (Van Rens et al., 2011) and include: (1) visual acuity < 0.30 logMAR (20/40 Snellen notation), and/or; (2) visual field < 30° around the central fixation point, and/or; (3) other severe visual field defects (i.e., hemianopia or cortical visual impairment), and/or; (4) visual acuity < 0.50 and an evident request for help because of limitations in activities of daily living (ADL) for which options in regular ophthalmic practice are not adequate, such as contrast sensitivity or glare.

Following a prior screening study in patients from these services (*N* = 1396) (Vreeken et al., 2014); patients were invited to participate in the trial if they were aged 50 years or older with self-reported hearing disability and in possession of HAs (mean pure tone thresholds at 1000, 2000 and 4000 Hz > 35 dB in the Netherlands, and >40 dB in Belgium). Patients without HAs were not invited to participate in the trial, i.e., due to time constraints it was not possible to wait for patients to have their HAs fitted and include them in the trial; the HA trial period usually takes months. Exclusion criteria were cognitive deficits, deafness and insufficient knowledge of the Dutch language. Potential participants received a comprehensive information letter and all participants signed informed consent before the start of the trial. In addition, participants were instructed to invite a communication partner to also participate in the study.

The sample size was based on expected progress on the primary outcome, which is the Communication Strategies subscale of the Communication Profile of the Hearing Impaired (CPHI) (Mokkink et al., 2010). It was estimated that 62 participants per arm were needed, when adjustment for clustering by eight strata and a 20% dropout rate were taken into account. This way a statistical power of 0.80 with an alpha of 0.05 (two-sided) was achieved and a relevant difference of 0.5 (Kramer et al., 2005) could be detected between arms.

The study was approved by the Medical ethical review committee of the VU University Medical Center (Amsterdam University Medical Centers) in the Netherlands and the Ethical Committee of University Hospitals UZ/KU Leuven in Belgium and conducted according to the principles of the Declaration of Helsinki. More details of the trial protocol are described elsewhere (Vreeken et al., 2013; Netherlands Trial Register, identifier: NTR2843).

### Measurements

Several standardized questionnaires were administered during the interviews at baseline and at 3 months follow-up. Measurements included main and secondary outcomes as well as other participant and health characteristics. During the interviews with the participant and at both time-points, communication partners were asked to fill out a paper-and-pencil questionnaire.

#### Participant and Communication Partner Characteristics

Background information of participants (including socio-demographic, vision, hearing and health characteristics) and communication partners (demographic characteristics) was collected as well as information on their relationship.

Data on the *visual impairment* included: decimal visual acuity which was extracted from the patient files from low vision rehabilitation centers, a question on self-reported visual disability from the National Eye Institute Visual Functioning Questionnaire (NEI VFQ-25), with response options ranging from excellent eyesight with both eyes to complete blindness (Mangione et al., 2001), self-reported ability to read newspaper headlines (yes or no), and the ‘Basic Aspects scale’ from the Dutch version Low Vision Quality of Life questionnaire (LVQOL) (Wolffsohn and Cochrane, 2000; van Nispen et al., 2010, 2011). The latter was rated as a summed score over five items with 6-point Likert response options (‘no’ to ‘not able’) about problems with seeing moving objects, eyes getting tired, watching television, glare and getting the right amount of light. The summed score was converted to a score between 0 and 100, with higher scores indicating more problems with basic aspects of vision.

Self-reported *hearing disability* was administered in the screening phase prior to the trial using three questions on situational hearing: ‘Can you follow a conversation with three to four people, without a HA?,’ ‘Can you follow a conversation with one person, without a HA?,’ and ‘Can you use a standard telephone?’ Weighted scores of the response options per item were “yes, without difficulty (1), yes, with slight difficulty (4), yes, with great difficulty (5), no, not able to (7),” which were summed into a total score between 3 and 21, with higher scores indicating more self-reported hearing loss (Smits et al., 2006; Vreeken et al., 2014).

*Speech recognition* in noise was assessed with Nationale Hoortest [NHT; National Hearing Test] (2004) which was installed on a laptop computer and performed with headphones^1^. The test presents three-digit sequences in a speech-spectrum noise background. The test was administered using an adaptive procedure. The signal-to-noise ratio (SNR) at which 50% of the digit sequences was recognized correctly is further referred to as the threshold. To categorize the hearing status of the participants, we used the categorization as proposed by Smits et al. (2006). Respondents with thresholds ≤−5.5 dB SNR were considered as having ‘good hearing,’ those with thresholds between −5.5 and −2.8 dB were considered having insufficient hearing and those with a poor hearing had thresholds >−2.8 dB SNR (Smits and Houtgast, 2005).

*Comorbidity* included the presence of seven most common somatic condition groups of chronic diseases: asthma or chronic obstructive pulmonary disease; cardiac disease; peripheral arterial disease; diabetes mellitus; cerebrovascular accident or stroke; osteoarthritis and rheumatoid arthritis and cancer.

*Health-related quality of life/health status* was measured with the generic self-report ‘utility’ questionnaire EQ-5D (Rabin and Charro, 2001). It assesses self-reported problems in five dimensions and at three levels each (no problems, some problems, a lot of problems): mobility, self-care, usual activities, pain/discomfort, and anxiety/depression. Utility scores are usually between 0 ‘death’ and 1 ‘perfect health.’ Scores below zero indicate a health state worse than death.

#### Primary Outcome Measure

The 35-item Dutch version of the CPHI consists of two domains: ‘Communication strategies’ and ‘Personal adjustment.’ The *Communication Strategies* domain of the CPHI assesses coping behavior in communicative situations related to hearing impairment and was considered the primary outcome of this study (Mokkink et al., 2010). This domain includes three subscales: Maladaptive Behaviors, Verbal Strategies, and Non-verbal Strategies. For all items of the CPHI, 5-point Likert scales are used which are divided by the number of items for every subscale. Mean scores are between 1 and 5, with lower scores indicating more problems.

#### Secondary Outcome Measures in DSL-Patients

*Personal adjustment* to hearing loss was measured with the Personal Adjustment domain of the CPHI. This domain investigates the feelings, attitudes and self-image which affect interpersonal relationships and is divided into the subscales: Self-acceptance, Acceptance of Loss, and Stress and Withdrawal. Similar to the Communication Strategies subscale, mean scores are between 1 and 5, with lower scores indicating more problems.

The ‘*Adjustment’* subscale of the LVQOL (Wolffsohn and Cochrane, 2000; van Nispen et al., 2010, 2011) was used to measure adjustment to vision loss, i.e., understanding of the eye condition, feelings of unhappiness or frustration and restrictions in visiting friends or family.’ The summed score over these four items with 6-point Likert response options was converted to a score between 0 and 100, with higher scores indicating more adjustment problems.

*Depressive* symptoms were measured with the Center for Epidemiological Studies - Depression Scale (CES-D) (Radloff, 1977) which consists of 20 items on depressive symptomatology experienced in the past week. Summed scores are between 0 and 60, where higher scores indicate more symptomatology and scores of 16 or higher indicate clinically significant depressive symptoms.

Feelings of *loneliness*, defined as social loneliness (discrepancy between the number of desired and actual social contacts) and emotional loneliness (quality of social contacts) were measured with the 11-item De Jong Gierveld Loneliness Scale (De Jong Gierveld and van Tilburg, 1999). In addition to a total score between 0 and 11, six items on emotional loneliness (scores 0–6) and five items on social loneliness (scores 0–5) were summed to calculate separate subscales, with higher scores indicating more loneliness.

The 10-item *Fatigue* Assessment Scale (FAS) was used to measure severity of fatigue (Michielsen et al., 2003, 2004). Summed scores on every 5-point Likert scaled item were between 10 and 50, with higher scores indicating more severe fatigue.

#### Secondary Outcomes in Communication Partners

*Attitudes of communication partners* toward the hearing impaired participant were assessed using the 10-item Hearing Handicap and Disability Inventory (HHDI) ‘Reactions of Others’ subscale (van den Brink, 1995; Kramer et al., 2005). It has 4-point Likert type scales which sum up to scores between 0 and 30, with higher scores indicating more problematic attitudes.

The *well-being of communication partners* of DSL-patients was evaluated with the Care-related Quality of Life - 7 Dimensions (CarerQol-7D) (Brouwer et al., 2006). The instrument assesses seven dimensions of caregiver burden, indicating ‘no,’ ‘some’ or ‘a lot of’ relational problems, mental health problems, problems combining daily activities with care, financial problems, physical health problems, fulfillment from caregiving and support with lending care. Dutch utility tariffs were used which offer weighted summary scores between 0 for the ‘worst’ and 100 for the ‘best’ caregiving situation (Hoefman et al., 2013).

### Data Preparation and Statistical Analyses

To prepare for data analysis, variables with skewed distributions were transformed using logarithmic (Ln) and square root transformations. In case of one or two missing items on the CES-D questionnaire, the missing value was replaced by the item-mean (the mean score on that item for all participants who completed that item) to be able to calculate the sum score and increase power (Bono et al., 2007).

Linear mixed models (LMM) were used to estimate treatment effects. Instead of using imputation techniques or deleting data as in complete case analyses, LMM allows inclusion of all data at baseline and follow-up (including data from participants who only have one measurement) which benefits the preciseness of the estimates. Beunckens et al. (2005) have shown that these ‘direct likelihood’ methods perform better with regard to outcome estimation in trials than other methods where data are imputed or deleted. Although we do report a baseline imbalance once, it was assumed in the LMMs that participants in both groups came from the same source population and that the means and variances were similar between groups. Therefore, we estimated one mean baseline score from which changes were calculated between groups over time for every outcome. The significance level was set at 0.05 (two-sided), however, since we performed multiple tests, we checked whether any effect found would hold at the 0.01 significance level. The initial analyses were performed according to the intention-to-treat (ITT) principle including all participants in the analyses as allocated. Effect sizes were estimated for the ITT analyses by calculating the standardized mean difference and 95% confidence intervals (CI) for every outcome (Morris, 2008) based on the crude baseline and follow-up mean differences and baseline standard deviations for every outcome. Effect sizes of 0.2 were considered to be small, 0.5 medium and 0.8 large (Cohen, 1988). In addition, analyses were performed with adjustment for gender and age and for other potential confounders (i.e., when a baseline difference between groups was found). Differential analyses were performed for age and gender to detect subgroup effects. For the secondary per-protocol analyses, participants who did not receive the intervention as assigned were excluded. Analyses were performed using SPSS 20 for Windows.

## Results

### Participants

**FIGURE 1:**
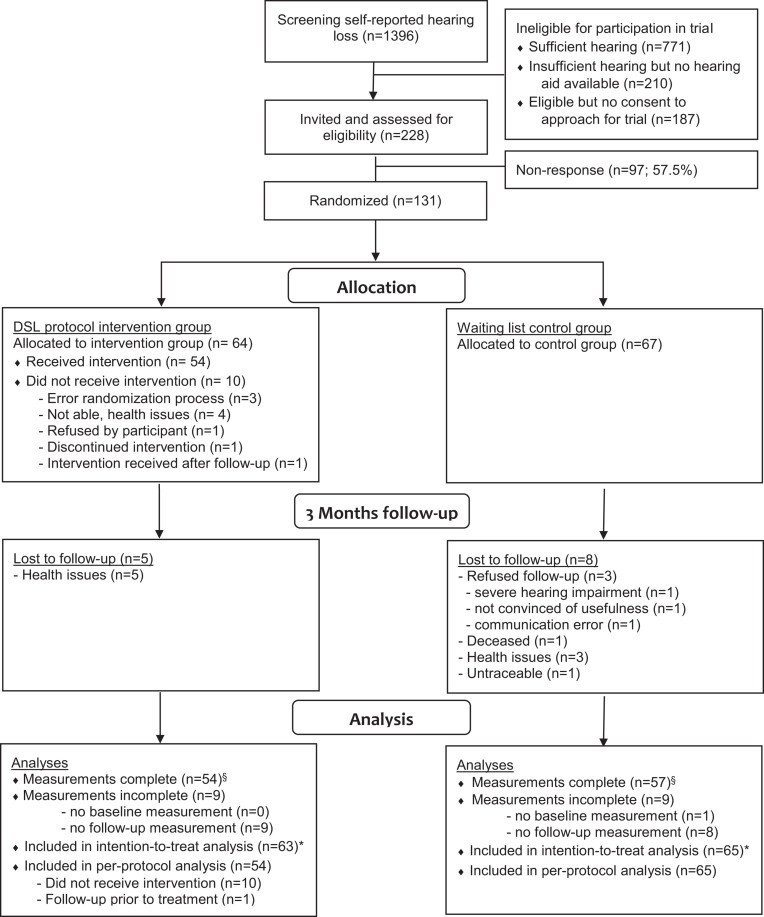
Flow of participants in the study comparing the DSL-protocol versus a waiting-list. ^∗^Participants who performed baseline measurements were included in the intention-to-treat analysis. Assuming they were missing at random, participants who discontinued during the study were kept in the analysis. In the per-protocol analysis, participants in the intervention group who did not receive the intervention as assigned were excluded from the analyses. ^§^ There were no significant differences between participants with complete and incomplete data.

Of the 228 eligible DSL-patients, 131 (57.5%) agreed to participate. The participant flow through the study is presented in Figure 1. Participants were randomly allocated to the intervention group (*N* = 64) or control group (*N* = 67). However, for several reasons, 12 participants allocated to the intervention group did not receive treatment. The median period between end of treatment and follow-up measurement was 49.5 days (interquartile range 32–69).

Table 1 presents the baseline characteristics of the study population. Half of the participants were female and had a mean age of 82 years. More than half of the participants lived alone as opposed to living together with a partner. Almost half of the sample had moderate to severe visual impairment in combination with poor hearing, whereas others had milder forms of functional loss in one or both senses. At baseline, significant differences between groups were found for gender, health-related quality of life and loneliness. There were relatively more women in the intervention group and their health-related quality of life was better compared to controls. In addition, a baseline imbalance was found on the loneliness scale, where the intervention group reported to be less lonely [mean 2.80 (SD 2.90)] compared to controls [mean 3.67 (SD 2.73); *p* = 0.035]. However, no significant baseline imbalances were found on the two loneliness subscales between trial arms.

**TABLE 1:** Baseline characteristics.

	Intervention (*N* = 64)	Control (*N* = 67)	*p*-Value	*n*
**Demographic**				
Age in years (mean (SD) [range])	81.3 (9.9) [56–97]	81.9 (10.0) [53–99]	0.71	131
Gender [*n* (%) female]	**27 (42.2**%)	**41 (61.2**%)	**0.03**	**131**
Partner status (% living alone)	33 (51.6%)	38 (57.6%)	0.49	130
Education in years, mean (SD) - Low - Medium/high	10.9 (3.2) 13 (23.2%) 43 (76.8%)	10.2 (3.6) 15 (24.2%) 47 (75.8%)	0.27	127
Income [*n* (%) financial constraints]	28 (44.4%)	26 (39.4%)	0.56	129
**Vision**				
Decimal visual acuity best eye, median [IQR] - Mild visual impairment ≥ 0.3 - Moderate visual impairment 0.1–0.3 - Severe visual impairment < 0.1	0.12 [0.05–0.3] 17 (27.4%) 22 (35.5%) 23 (37.1%)	0.18 [0.08–0.32] 21 (33.9%) 25 (40.3%) 16 (25.8%)	0.39	124
Newspaper headlines (% not able to read)	28 (43.8 %)	39 (59.1%)	0.08	130
Basic aspects (LVQOL [0–100])^†^, mean (SD)	50.7 (18.7)	52.5 (20.0)	0.61	121
Self-report (NEI VFQ-25 item) - Excellent - Good - Moderate - Bad - Very bad - Completely blind	0 (0%) 0 (0%) 9 (14.1%) 28 (43.8%) 24 (37.5%) 3 (4.7%)	0 (0%) 0 (0%) 16 (24.2%) 26 (39.4%) 19 (28.8%) 5 (7.6%)	0.38	130
Causes of vision loss^∼^ - Macular degeneration - Cataract - Glaucoma	37 (57.8%) 21 (32.8%) 10 (15.6%)	40 (59.7%) 18 (26.9%) 9 (13.4%)	0.86 0.46 0.72	131 131 131
**Hearing**				
NHT, median [IQR] dB SNR^‡^ - Good ≤ −5.5 dB SNR - Insufficient −5.5 to −2.8 dB SNR - Poor > −2.8 dB SNR	−1.6 [−4.1 – 1.9] 5 (7.9%) 19 (30.2%) 39 (61.9%)	−0.6 [−3.1 – 1.6] 5 (7.7%) 13 (20.0%) 47 (72.3%)	0.40	128
Self-report [3–21], mean (SD)^†^	14.3 (3.9)	15.4 (3.3)	0.10	129
**Dual Sensory Loss**				
Severity of DSL - Mild VI, good/insufficient hearing - Mild VI, poor hearing - Moderate/severe VI, good/insufficient hearing - Moderate/severe VI, poor hearing	14.8% 11.5% 24.6% 49.2%	11.7% 23.3% 18.3% 46.7%	0.37	121
Onset of sensory loss - First hearing loss - First vision loss	25 (39.1%) 39 (60.9%)	32 (50.8%) 31 (49.2%)	0.18	124
Duration DSL (years), median [IQR]	4.0 [2–7]	5.5 [2.75–10]	0.33	129
**Health**				
Health-related QOL (EQ-5D), mean (SD)^#^	**0.73 (0.26)**	**0.63 (0.29)**	**0.05**	**130**
Comorbidity, mean (SD)	1.8 (1.3)	2.2 (1.4)	0.09	131

*Bold: Significant difference between intervention and control group (p < 0.05). ^†^Higher scores indicate more problems. ^‡^NHT scores range between approximately −10 (the best normally hearing individual) to +4 dB SNR; lower scores indicate better speech discrimination (Smits et al., 2006). ^#^Lower scores indicate more problems. ^∼^Because of not excluding categories, the sum may exceed 100%. SD, standard deviation; IQR, inter-quartile range; DSL, Dual Sensory Loss; LVQOL, Low Vision Quality of Life questionnaire; NEI VFQ-25, National Eye Institute Visual Functioning Questionnaire; NHT, National Hearing Test; dB SNR, signal-to-noise ratio in decibel; QOL, quality of life; EQ-5D, EuroQol 5-dimensions.*

Table 2 presents baseline characteristics of the communication partners. For four participants, baseline and follow-up questionnaires were filled out by different communication partners; they were analyzed separately. In total, data was collected from 113 communication partners (baseline *n* = 103; DSL *n* = 52; control *n* = 48), follow-up (*n* = 84; DSL *n* = 43; control *n* = 41). For three baseline questionnaires the corresponding participant was missing and therefore the allocation was unknown, however, their baseline data were included in the analyses. Half of the communication partners were the spouse, and approximately one third were the child of the participant. More than half were living together with the participant and most communication partners were women. There were no baseline imbalances on the demographic characteristics or outcome measures between trial arms. At baseline, the attitude and wellbeing of communication partners as measured with the HHDI and CarerQol-7D seemed adequate.

**TABLE 2:** Main characteristics of communication partners at baseline.

	Intervention *n* = 57	Control *n* = 53	Total *n* = 113^‡^	*p*-Value	*n*
**Demographic**					
Age (bimodal distribution)	66.7 (13.4)	66.5 (16.4)	66.3 (14.8)	0.936	110
Gender (women)	43 (75.4%)	38 (71.7%)	82 (72.6%)	0.672	110
Relationship with patient* - Spouse - Brother/sister (in law) - Child - Other	27 (50.9%) 2 (3.8%) 18 (34.0%) 6 (11.3%)	26 (53.1%) 1 (2.0%) 12 (24.5%) 10 (20.4%)	53 (50.5%) 4 (3.8%) 32 (30.5%) 16 (15.2 %)	0.494	102
Living with DSL-patient* (yes)	29 (52.7%)	28 (54.9%)	57 (52.3%)	0.848	106

*Eight, four, eleven, and seventeen missing values for relationship, living situation and baseline HHDI and CarerQol-7D, respectively. ^‡^Treatment allocation was unknown for three baseline questionnaires, however, the data was included in the ITT-analyses (control group). SD, standard deviation; DSL, Dual Sensory Loss; HHDI, Hearing Handicap and Disability Inventory; CarerQol-7D, Care-related Quality of Life - 7 Dimensions questionnaire.

### Treatment Effects

Intention-to-treat analyses showed an effect on CPHI – Verbal strategies in favor of the control group, which did not remain significant after adjustment for (age, sex and) health-status. No statistically significant effects of the DSL-protocol were found in none of communication or wellbeing outcomes compared to waiting-list controls in the patient sample (Table 3). Per protocol analyses showed similar results (data not shown). The DSL-protocol showed a significant positive effect on attitudes of communication partners compared to wait-list controls as measured with the ‘HHDI Reactions of Others’ scale; the intervention group remained stable, whereas the control group slightly deteriorated. This effect did not remain significant at a 0.01 significance level. Although differences in health status and gender differences between groups influenced the outcomes for the separate groups to some extent, this was not reflected in the adjusted treatment effects. No overall effects were found on the Adjustment subscale of the LVQOL, however, one differential effect was found, where women in the intervention group seemed to benefit more from the intervention than men in the intervention group (*p* = 0.010; data not shown).

**TABLE 3:** Effects of Dual Sensory Loss protocol on communication and psychosocial health in patients and their communication partners.

	Baseline		3-months follow-up												
	Intention-to-treat									Intention-to-treat adjusted					
										Sex/age		Health status		Sex/age/health status	
		*n*		*n*		*n*	Δ	*p*-Value	Effect-size [95% CI]^ 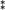 ^	Δ	*p*-Value	Δ	*p*-Value	Δ	*p*-Value
*Primary outcomes*															
Communication strategies (CPHI)^#^															
- Maladaptive behavior [1–5]*^#^	4.4	130	4.5	55	4.5	59	0.02	0.76	0.00 [−0.34, 0.34]	0.01	0.92	0.01	0.89	0.01	0.93
- Verbal strategies [1–5]^¶#^	2.6	130	2.5	55	2.7	59	**0.17**	**0.04**	**0.60 [0.25, 0.95]**	**0.20**	**0.04**	0.16	0.06	0.16	0.07
- Non-verbal strategies [1–5]^¶#^	3.3	130	3.2	55	3.3	58	0.09	0.53	0.29 [−0.06, 0.63]	0.13	0.48	0.12	0.52	0.13	0.48
*Secondary outcomes*															
Personal adjustment to hearing loss (CPHI)^#^															
- Self acceptance [1–5]*^#^	4.4	130	4.5	55	4.5	58	0.02	0.92	−0.05 [−0.39, 0.30]	−0.00	0.96	−0.01	0.89	−0.01	0.93
- Acceptance of hearing loss [1–5]^¶#^	3.2	130	3.4	55	3.3	58	−0.16	0.22	−0.23 [−0.57, 0.12]	−0.16	0.22	−0.18	0.18	−0.18	0.18
- Stress and withdrawal [1–5]^¶#^	3.3	130	3.4	55	3.4	58	0.01	0.95	−0.01 [−0.35, 0.33]	0.00	0.95	−0.01	0.90	−0.02	0.88
Adjustment to vision loss (LVQOL [0–100])^‡⁣†^	23.4	128	25.5	55	27.7	57	−2.21	0.54	0.06 [−0.28, 0.41]	−2.12	0.55	−2.00	0.58	−2.29	0.58
Depression (CES-D [0–60])^‡†^	9.8	130	10.0	55	10.1	58	−0.13	0.85	−0.05 [−0.39, 0.30]	−0.08	0.93	−0.62	0.63	−0.51	0.70
Fatigue (FAS [10–50])^§†^	20.3	128	21.3	55	21.1	58	0.21	0.95	−0.03 [−0.38, 0.32]	0.00	0.99	−0.45	0.73	0.24	0.81
Loneliness (De Jong Gierveld [0–11])^‡†^	2.8	130	3.4	55	2.7	58	0.64	0.08	0.29 [−0.06, 0.63]	0.67	0.07	0.65	0.12	0.69	0.11
- Emotional loneliness [0–6]^‡†^	1.8	130	2.1	55	1.8	58	0.31	0.18	0.20 [−0.15, 0.54]	0.34	0.14	0.32	0.26	0.36	0.21
- Social loneliness [0–5]^‡†^	1.0	130	1.1	55	0.9	58	0.20	0.28	0.30 [−0.05, 0.64]	0.67	0.07	0.22	0.30	0.22	0.37
**Communication partners**															
HHDI - Reactions of others [0–30]^†^	1.5	100	1.5	43	1.7	41	**0.13**	**0.02**	0.12 [−0.27, 0.51]	**0.13**	**0.02**	n.a.	n.a.	n.a.	n.a.
CarerQol-7D [0–100]^#^	83.1	93	86.9	37	83.7	77	−3.23	0.15	−0.17 [−0.58, 0.23]	−3.16	0.17	n.a.	n.a.	n.a.	n.a.

*Bold: Significant treatment effect (p < 0.05). Data transformations ^¶^x−1, *ln(6−x), ^‡^√(x + 1) and ^§^ln(x) were used for analyses and retransformed. ^†^Higher scores indicate more problems. ^#^Lower scores indicate more problems. ^Δ^Difference between groups over time, a negative value means a positive effect for the intervention group. ^
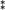
^ A negative standardized mean difference means a positive effect size in favor of the intervention group; as SMDs were estimated from crude means and SDs, the direction of the intervention effect may sometimes deviate from Δ. DSL, Dual Sensory Loss; CPHI, Communication Profile of the Hearing Impaired; LVQOL, Low Vision Quality of Life questionnaire; CES-D, Center for Epidemiological Studies – Depression Scale; FAS, Fatigue Assessment Scale; HHDI, Hearing Handicap and Disability Inventory; CarerQol-7D, Care-related Quality of Life - 7 Dimensions questionnaire; n.a., not applicable.*

## Discussion

The purpose of this study was to investigate the effectiveness of a DSL-protocol on the use of effective communication strategies and wellbeing in patients and their communication partners. In this randomized controlled trial no significant treatment outcomes were found in DSL-patients. The effects that were found regarding verbal strategies and loneliness indicated slightly more favorable outcomes for the control group. In turn, a treatment effect was found in communication partners: their attitudes toward the DSL-patients remained stable, whereas controls slightly deteriorated.

Even after having performed multiple statistical tests due to a relatively large number of study outcomes which by chance could have resulted in a positive effect, there were hardly any effects of the intervention, making a type II error an unlikely explanation for our findings. The results are in line, however, with other studies on interventions for older adults with vision and/or hearing loss. Two recent studies on integrated care for older adults with DSL showed no difference in hearing handicap or quality of life (McMahon et al., 2017) and only on one (instrumental activities of daily living) of four domains of social participation (Roets-Merken et al., 2018). In addition, a meta-analysis of eight interventions for older adults with vision or hearing loss revealed no significant effects in favor of interventions on quality of life or daily functioning (Roets-Merken et al., 2015). Also a program for partners of visually impaired older adults showed, with the exception of two items on awareness, no significant improvement in understanding of low vision, confidence to deal with low vision, self-efficacy or emotional well-being (Larizza et al., 2011). Finally, a recent modified web-based version of the home based communication program for people with hearing loss on which the DSL-protocol was based, called the SUpport PRogram (SUPR) for HA users aged 50+, also showed no effect on communication strategies, but only on short and long-term HA use and satisfaction (Meijerink et al., 2020).

There are some aspects of the intervention, participant characteristics and the study design that might have contributed to the unexpected lack of positive results in the intervention group, shown in this study. First, perhaps the DSL-protocol is not adequate to change behavior of patients in a short period of time. The number of appointments between OTs and patients might have been insufficient for the extensive amount of information addressed in the protocol. A larger number of sessions would provide the opportunity to repeat information and exercise allows the patient to go through all stages of behavior change. In addition it will create more room for involvement of communication partners. Although this adaptation might improve outcomes of the intervention, providing more sessions must be feasible for society in terms of time and costs. Possibly, the few therapy sessions may make patients more aware of their communication problems which explains the negative effects found in this study, but the treatment was insufficient to influence behavior and the impact on health outcomes.

Secondly, during the design phase of the DSL-protocol, we considered it to be a strength that one single health care professional (i.e., an OT) would deliver the entire DSL-protocol, as it enables patients and professionals to build a trusting relationship. OTs performed the first two ‘practical’ chapters on HAs, other assistive devices and home environmental adaptations, as well as the third chapter on communication strategies and psychosocial functioning. The latter involved discussing communication issues with patient and partner, teaching new skills, changing existing behaviors and addressing psychosocial issues. However, in hindsight OTs might not have been the best skilled professionals for this chapter despite the 1 day training they received. OTs are generally ‘practically oriented,’ some OTs debriefed that they did not feel comfortable giving advice on communication and psychosocial issues. For example the older adults with DSL in our study seem to perceive more depressive feelings compared to older adults in the general population (Brailean et al., 2016). This indicates that the different components may not have been optimally executed, and may explain the lack of finding an effect. A professional from a different discipline (e.g., social worker) may have been a better choice to perform this part of the protocol. These notions are in line with an interview study by Fraser et al. (2019) who found that, regardless their backgrounds, professionals working with adults with DSL reported that they regularly fulfilled additional roles for which they felt they were not trained, such as helping the person and their families with depression and acceptance, having multiple consultations and help with navigation through the health system. They suggested more education for themselves and for family members of adults with DSL (Fraser et al., 2019). Further research is necessary to investigate how the protocol might be adapted or whether it is necessary to involve social workers or other mental health care professionals in DSL-care or offer more training from a systems perspective.

Thirdly, ceiling and floor effects on some subscales indicating a lack of room for improvement after baseline – e.g., loneliness scores did not indicate problematic behavior – may have hampered the possibility to measure treatment effects, and the slight deterioration in the treatment group may be considered an accidental finding. On the other hand, as compared to the control group, the DSL-protocol may have instigated awareness by the DSL-patient of their current situation and a subsequent response shift in measurements at follow-up as reflected in deteriorations in feelings of loneliness and verbal strategies. As the decline may be temporary, this hypothesis should be tested in future studies with more intensive treatment, monitoring and measurement of maintenance effects. In the former study by Kramer et al. (2005), who developed a communication program for people with hearing loss only and on which our DSL-protocol was based, the direction of effects of the program differed depending on the measurement time-point, directly post intervention or at 6 months, and the outcome measure. It may take more time to let all the information and advice sink in, to get used to and practice newly learned skills and change behavior. However, the time between the last session and the follow-up measurement was approximately one and a half months, which may be too short. In addition, a floor effect was also found on the attitudes toward DSL-patients of communication partners (HHDI Reaction of others subscale) with the intervention group remaining stable, which was also observed in a study by Kramer et al. (2005).

Fourthly, the choice of the study population may also have influenced the results. All participants in the present study were experienced HA users with well-established habits and behaviors. As changing old habits is difficult, the protocol might have a greater effect in case the protocol would have been performed at the time of purchase of the first HA. This may have been a more suitable moment, because it is when patients acknowledge their hearing impairment and are in the process of adapting to the new situation, they may be more open to learn new skills and behaviors. In research by Kramer et al. (2005) a significant interaction was found between groups as an effect of their communication program, showing that first time users performed better on communication strategies compared to experienced HA users. However, this assumption could not be examined in the present study as no first time users were included due to pragmatic reasons (i.e., limitations in time and study resources). The recruitment of the study population might also explain the results of the present study. Participants in the present study were recruited through low vision services instead of audiology care in other studies investigating communication programs (Chisolm et al., 2004; Kramer et al., 2005). Presumably, audiology care patients experience more severe hearing-related problems compared to their peers recruited from low vision, otherwise they would not have sought audiological help. The present study was supply-oriented, participants were not actively searching for help with communication. The living situation of the participants could be another factor as relatively many participants (more than half) lived alone and might only rarely be in complex listening situations involving more people. Possibly, participants did not need to use or change communication strategies. Both assumptions could explain the relatively good baseline scores on the CPHI-questionnaire compared to the reference scores (Mokkink et al., 2010). Generally, participants were at a very advanced age. OTs gave advice on how to use effective communication strategies and cope with DSL, however, not all participants and partners put the effort in it to take action and change their behavior. Some participants experienced other more urgent health issues, lacked the assertiveness needed, or did not feel like changing their behavior and said they felt too old.

Finally, in contrast to the participants, the inclusion of communication partners was not strictly controlled as it was not the main focus of our study. Besides not all participants had a communication partner willing to participate, there were also some changes during the study period in which the communication partner took part in the study; baseline and follow-up measurements were sometimes administered by different communication partners.

In addition to the abovementioned limitations of the study, there are also a number of strengths. Firstly, the design of the study minimizes bias, i.e., selection and detection bias. In this randomized controlled trial participants were randomly allocated to the intervention or control group and the investigator conducting the analyses as well as the research assistants administering the interviews where masked for treatment allocation. Secondly, the screening on hearing problems performed prior to the trial is considered a strength. Hearing loss develops into hearing impairment which, in turn, drastically increases the risk of social engagement restrictions and emotional deficits (Gopinath et al., 2012). As all participants in the study experienced hearing impairment, the study was targeted at a unique group of vulnerable patients. Lastly, conversation involves both communicators and the involvement of communication partners is an important third strength of the DSL-protocol.

## Conclusion

In conclusion, no significant improvements of communication or psychosocial functioning in DSL-patients were found in the present study. However, the attitudes of communication partners toward DSL-patients remained stable whereas the control group deteriorated. Still, this study has yielded many new insights into the different aspects of having DSL, the needs of patients and communication partners and the study design. Therefore, further study toward care for this vulnerable group of older adults and to investigate how the still promising DSL-protocol may be adapted to reach its full potential, is warranted.

## Data Availability Statement

The raw data supporting the conclusions of this article will be made available by the authors, without undue reservation.

## Ethics Statement

The studies involving human participants were reviewed and approved by Medical Ethical Review Committee, Amsterdam University Medical Centers, Amsterdam, Netherlands and Ethical Committee, University Hospitals UZ/KU, Leuven, Belgium. The patients/participants provided their written informed consent to participate in this study.

## Author Contributions

RN, SK, and GR originally designed the study. RN was the main applicant of the research grant. HV developed the DSL-protocol, performed data collection and data analysism and wrote the manuscript which was approved by all authors. RN helped interpret the outcomes.

## Conflict of Interest

The authors declare that the research was conducted in the absence of any commercial or financial relationships that could be construed as a potential conflict of interest.

## Footnotes

1 www.thenationalhearingtest.org

## Abbreviations

CPHI: Communication Profile for the Hearing Impaired
CarerQol-7D: Care-related Quality of Life - 7 Dimensions
CES-D: Center for Epidemiological Studies - Depression Scale
DSL: Dual Sensory Loss
EQ-5D: EuroQol 5-Dimensions
FAS: Fatigue Assessment Scale
HA: hearing aid
HHDI: Hearing Handicap and Disability Inventory
ITT: Intention-To-Treat
LMM: linear mixed models
LVQOL: Low Vision Quality of Life questionnaire
NEI VFQ-25: National Eye Institute Visual Functioning Questionnaire - 25 items
NHT: National Hearing Test
NTR: Netherlands Trial Register
OT: occupational therapist
PP: per-protocol
SNR: signal-to-noise ratio
SPSS: Statistical Package for the Social Sciences.
